# Time-Optimal Trajectory Planning Method for Servo PMSM Based on Short-Term Dynamic Feasible Region Constraint

**DOI:** 10.3390/s26134010

**Published:** 2026-06-24

**Authors:** Hui Li, Jianfu Li, Xuewei Xiang, Peng Jiang, Bin Yuan, Renkuan Liu

**Affiliations:** 1 State Key Laboratory of Power Transmission Equipment Technology, School of Electrical Engineering, Chongqing University, Chongqing 400044, Chinaxueweixiang@cqu.edu.cn (X.X.); jiangpeng@stu.cqu.edu.cn (P.J.); 2 Dongfang Electric Wind Power Co., Ltd., Deyang 618000, China; cqubingo@163.com; 3 Dongfang Electric Digital Technology Co., Ltd., Chengdu 610218, China; uestclrk@uestc.edu.cn

**Keywords:** servo PMSM, trajectory planning, jerk and time-optimal, dynamical constraints

## Abstract

Aiming at addressing the problem whereby the traditional time-optimal trajectory planning based on the steady-state torque–speed characteristic cannot fully exploit the short-term dynamic output performance of the servo permanent magnet synchronous motor (SPMSM), a time-optimal trajectory planning method for the SPMSM based on the short-term dynamic feasible region constraint is proposed to effectively improve the response speed. Firstly, the dynamic trapezoidal domain operation boundary is obtained by analyzing the motor working point variation curve and considering factors such as the working temperature and trajectory control, which constitutes the torque–speed value and the dynamic constraint mechanism of trajectory planning. Secondly, based on the energy consumption model, the average thermal power is used to represent the torque overload limit condition, and a dynamic constraint method based on the short-term dynamic torque–speed operation boundary is proposed. Then, in order to reduce the computational load in the online millisecond-level response, a time-optimal trajectory optimization algorithm based on sequential least squares is proposed to calibrate the positioning time of the time-optimal trajectory under different working temperatures and angles. Finally, a simulation and experimental comparisons of the time-optimal trajectories under different angles and working temperatures are carried out to verify the effectiveness of the proposed method.

## 1. Introduction

Servo permanent magnet synchronous motors (SPMSMs) are widely used in servo equipment such as radar electro-optical sensors and industrial robots due to their excellent performance [1,2,3]. In short-term rapid positioning conditions, servo equipment requires SPMSMs to respond quickly to the target angle. Time-optimal trajectory planning can effectively reduce the positioning time of the SPMSM to reach the target angle, and the shortest positioning time is mainly determined by the dynamic constraints of the maximum speed, acceleration, and shock value. The maximum shock value can be set according to the load operation characteristics of the servo system, while the maximum speed and acceleration are often selected based on the steady-state torque–speed values of the SPMSM. However, the steady-state torque–speed dynamic boundary conditions are unable to accurately represent the short-term dynamic feasible region of the torque and speed of the SPMSM under short-term high overload, a wide temperature range, and non-periodic transient conditions, thus failing to fully utilize the short-term output performance of the SPMSM. Considering the operation characteristics of SPMSMs in short-term rapid positioning conditions, taking into account the short-term dynamic feasible region constraints of SPMSMs and conducting time-optimal trajectory planning is the key to improving the rapid response capabilities of SPMSMs.

In terms of time-optimal trajectory planning, existing research often relies on various trajectory types, such as trapezoidal, S-curve, polynomial, and spline curves [4,5,6,7,8], using the maximum values of speed and acceleration to represent the motor boundaries as dynamic constraints [9] for time optimization in positioning. However, the trapezoidal trajectory’s step change in acceleration leads to problems such as position overshoot and mechanical wear [10]. The S-curve reduces the impact of the motion process by introducing a transition process and has been widely applied in servo systems with certain smoothness requirements [11,12]. Bai and Lu et al. have conducted time-optimal trajectory planning optimization under kinematic and dynamic constraints based on the S-curve [13,14]. To address the issue of discontinuous jerk in traditional S-curve trajectory planning, Rupesh et al. [15] introduced a novel critical motion parameter method to appropriately tune the 15-segment fourth-order symmetrical S-curve profile to achieve low-vibration P-T-P motion in a flexible system. Yi Fang et al. adopted an S-shaped function to establish trajectory planning with adjustable shock phase duration parameters, making the generated trajectory infinitely continuously differentiable under given constraints [16]. Some studies have also adopted asymmetric S-curve trajectories in the acceleration and deceleration phases to shorten the operation time [17]. Polynomial trajectories are widely used in actual position servo systems due to their simple expression functions and adjustable order according to the actual smoothness requirements, especially in high-precision application scenarios, where high-order polynomial trajectory planning can achieve smooth and high-precision servo positioning [18]. Yue Ming et al. [19] achieved the smooth and rapid operation of track vehicles during lane changes based on quintic polynomial trajectory planning; Lee et al. [20] proposed an optimization method for transport robots using polynomial trajectories and a method to determine the optimal order of polynomials under specified working conditions. Based on different types, intelligent optimization algorithms can be used to optimize trajectories with time as the objective [21,22,23,24].

However, although the above researchers have carried out time-optimal design for various types of trajectory planning, when setting the dynamic constraints of the SPMSM, except for the maximum impact value, they all essentially use the maximum values of the speed and acceleration to represent the performance boundary of the motor output. However, in actual operation, only the trapezoidal trajectory will pass through the working points corresponding to the maximum speed and acceleration. When the SPMSM operates under continuous acceleration trajectories, it will not pass through this working point, thus not fully utilizing the torque and speed output performance of the SPMSM. Moreover, the torque and speed operating boundaries of the SPMSM will also change dynamically under different loads and working temperatures. The traditional dynamic constraint method based on setting the maximum torque and speed values under long-term steady-state conditions and then determining the maximum acceleration and speed values is unable to accurately represent the short-term dynamic feasible domain boundary of the SPMSM. Therefore, further research is needed regarding the optimization design of the time-optimal trajectory of the SPMSM under short-term dynamic feasible domain constraints, so as to fully exploit its short-term overload output performance. The optimization design based on the control strategy can also effectively improve the tracking effect of the optimal trajectory in terms of time [21,22], but the literature still lacks sufficient exploration of the motor performance.

In view of the above issues, a time-optimal trajectory planning method based on short-term dynamic feasible region constraints is proposed to fully utilize the short-term overload output performance of the SPMSM. The main contributions of this paper are as follows: (1) to address the problem whereby traditional dynamic constraints based on steady-state parameters are unable to fully exploit the rapid response capabilities of SPMSMs, a dynamic constraint method for torque and speed under short-term overload is proposed; (2) considering the kinematic and dynamic constraints under actual operating conditions, a time-optimal trajectory optimization algorithm based on the sequential least squares method is proposed, and the shortest positioning time under different temperatures and positioning angles is determined.

## 2. Trajectory Planning Dynamic Constraint Conditions

### 2.1. Kinematic Constraints of Typical Trajectories

For point-to-point position servo systems, the most commonly used time-optimal trajectory planning includes trapezoidal curves, S-shaped curves, polynomials, etc. To analyze the mechanisms of the dynamic constraints on various trajectories, taking the maximum positioning stroke of the radar photoelectric sensor in this study, which is 180°, as an example, and using trapezoidal curves, S-shaped curves, and quintic polynomial trajectories, the positioning time is compared under the same dynamic and kinematic constraints. The curves of the speed, acceleration, and impact are shown in Table 1.

**Figure 1:**
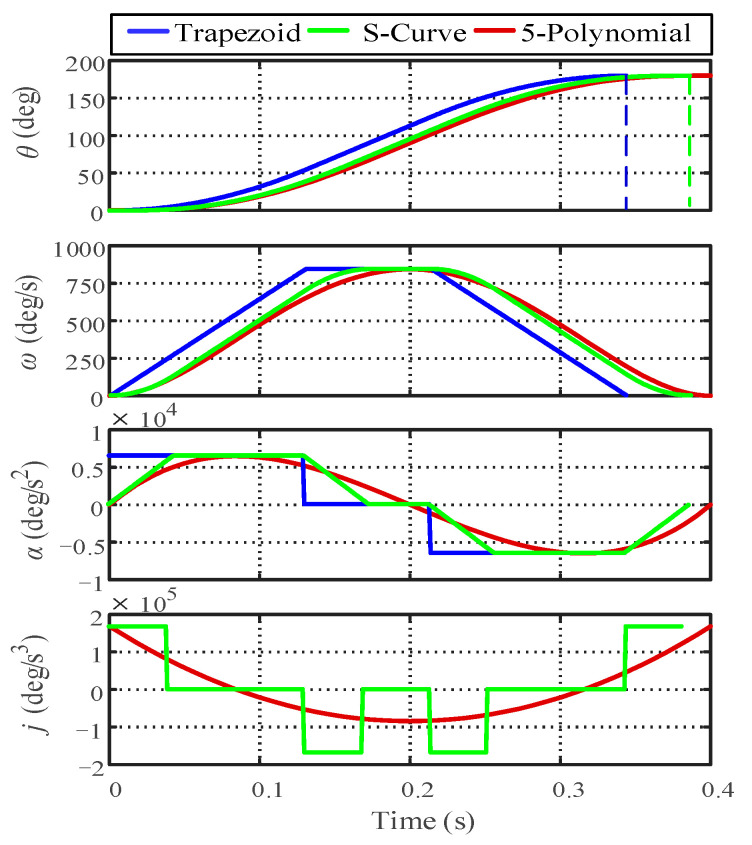
Comparison of positioning times for common trajectories under the same dynamic constraints.

Under the same kinematic and dynamic boundary constraints, the positions, velocities, accelerations, and shock waveforms of the three groups of trajectories are as shown in Figure 1. From the figure, it can be seen that, under the same positioning angle and dynamic constraints, the positioning times of the trapezoidal, seven-segment S-curve, and quintic polynomial trajectories increase successively, being 0.35 s, 0.38 s, and 0.4 s, respectively. Although the trapezoidal trajectory has the shortest positioning time, its acceleration step change leads to an infinite shock. The shock of the seven-segment S-curve trajectory changes in a maximum-value step, and the discontinuity of the shock will bring adverse effects such as vibration impacts, noise, wear, and precision loss to the servo system. Meanwhile, the quintic polynomial, despite having the longest positioning time under the smoothness requirement, can maintain continuous shock changes throughout the operation.

**Figure 2:**
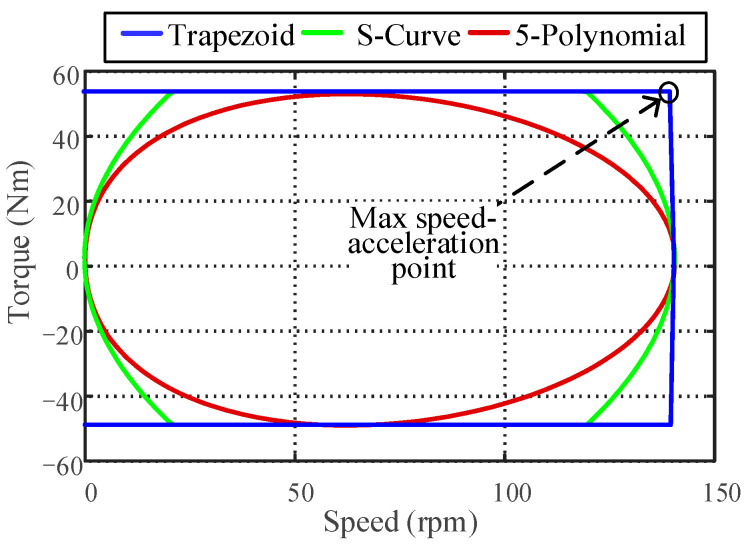
The distribution of the torque–speed working curves under 3 sets of trajectories.

To analyze the mapping relationships among the maximum-value constraints of the dynamic velocity and acceleration in the motor torque–speed operation domain, the working curves of the above three groups of trajectory planning are plotted, as shown in Figure 2.

As can be seen from Figure 2, the trapezoidal trajectory passes through the torque–speed operating point corresponding to the maximum speed and acceleration values; the seven-segment S-curve, due to the limitation of the maximum impact value, does not pass through the maximum torque–speed operating point, and only some curve segments reach the rectangular boundary. Meanwhile, the elliptical working curve of the quintic polynomial trajectory only has two points reaching the rectangular boundary throughout the positioning process, thus resulting in a longer positioning time than for the trapezoidal and S-curve trajectories under the same constraints. Therefore, it can be determined that the torque–speed rectangular domain formed by the maximum speed and acceleration values places the working curve of any trajectory within the envelope range.

**Figure 3:**
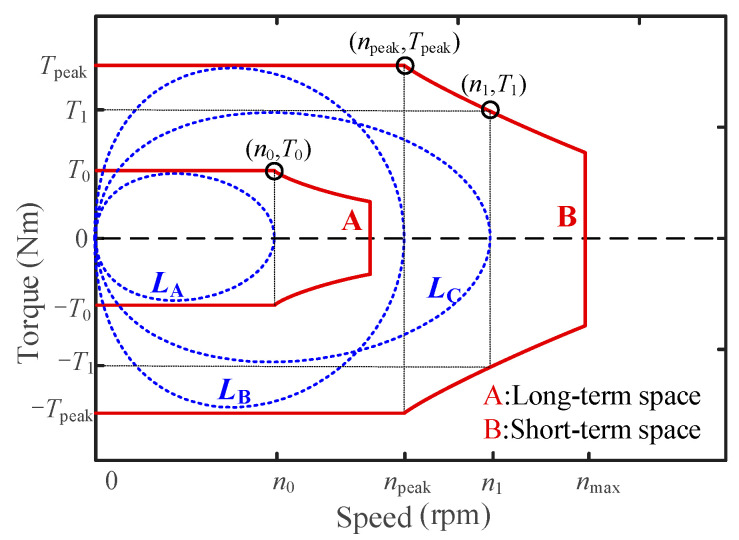
Schematic diagram of the selection of the kinematic constraint rectangular boundary.

Traditional research on time-optimal trajectory planning, without integrating detailed motor electromagnetic design parameters, is unable to obtain accurate output characteristic curves for SPMSMs. At this time, the maximum values of the speed and acceleration are often set based on the rated speed–torque (*T*_0_, *n*_0_) or peak speed–torque (*T*_peak_, *n*_peak_) parameters given on the motor nameplate. Meanwhile, according to the different duty cycles, motors can be divided into long-term and short-term working zones of torque–speed, corresponding to the temperature rise design requirements when reaching the thermal steady state under long-term operation and the temperature rise not exceeding the limit under short-term operation. An illustration of the above torque–speed dynamic constraints in the motor operation domain is shown in Figure 3. *T*_1_ and *n*_1_ represent a working point during the constant-power stage.

From Figure 3, it can be seen that the dynamic constraints for selecting the maximum speed and acceleration based on the rectangular operating domain are set to improve the utilization rate of the motor output torque. To achieve this, the turning point between the constant-torque zone and the constant-power zone is often used for the maximum acceleration and speed values, such as the rated operating point (*n*_0_, *T*_0_) and the peak operating point (*n*_peak_, *T*_peak_). At this time, the torque output of the rectangular operating domain can be maximized within the long-term working area A or the short-term working area B, as shown in the working curves *L*_A_ and *L*_B_ in the figure. This is also effective in reducing the trajectory positioning time for servo scenarios requiring a higher speed during the positioning process, as shown in the working curve *L*_C_ in the figure.

### 2.2. Dynamic Boundary Based on the Trapezoidal Feasible Region

**Figure 4:**
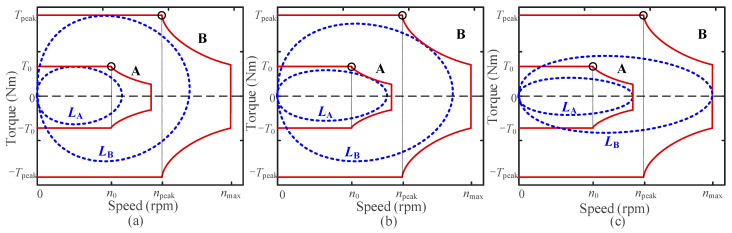
Diagrammatic analysis of the time-optimal trajectory’s working curve reaching the kinematic limit boundary. (**a**) Maximum torque constraint; (**b**) Maximum power constraint; (**c**) Maximum speed constraint.

The dynamic boundary constraints based on the rectangular feasible region do not fully utilize the torque–speed feasible region of the SPMSM below the constant-power curve. Therefore, this study analyzes the extreme shortest positioning time of the SPMSM within the actual trapezoidal feasible region constraints of the photoelectric hoist on the basis of the traditional trajectory planning dynamic constraints. In this section, for the impact continuous trajectory, using the corresponding torque–speed working curve distribution of the time-optimal trajectory, the graphical method is adopted to analyze the limit tangency situation between it and the actual torque–speed trapezoidal boundary of the SPMSM. According to the different triggering of the maximum torque, the constant-power curve, and the maximum speed constraints, we proposed three basic types of limit constraint situations. The graphical analysis is shown in Figure 4.

From Figure 4, it can be seen that, at the limit of the shortest positioning time, the torque–speed working curve of the impact continuous trajectory is tangent to the dynamic boundary. As seen in Figure 4a, the working curve triggers the maximum torque constraint, corresponding to a servo system with a higher required torque; as seen in Figure 4b, the working curve triggers the constant-power operation boundary constraint; as seen in Figure 4c, the working curve triggers the maximum speed constraint, corresponding to a servo system with a higher required speed. For these three cases, two different working modes are also considered for the limit constraints. It is evident that, based on the boundary constraint of the trapezoidal feasible region of torque–speed, the working curve’s speed can exceed the boundary of the traditional rectangular region, thereby shortening the positioning time.

### 2.3. Analysis of the Influencing Factors of the Feasible Region Boundary

The output speed–torque of the SPMSM is mainly influenced by factors such as the DC bus voltage, control strategy, inverter, electromagnetic parameters, and heat dissipation capacity. The motor transient stator voltage equation of a PMSM in the *d*-*q* rotating coordinate system is

(1) $$\left\{\begin{array}{l} u_{d}=R_{s}i_{d}+L_{d}\frac{di_{d}}{dt}-\omega_{e}L_{q}i_{q}\\ u_{q}=R_{s}i_{q}+L_{q}\frac{di_{q}}{dt}+\omega_{e}L_{d}i_{d}+\omega_{e}\psi_{f} \end{array}\right.$$

Among the terms, *u*_d_ and *u*_q_ represent the stator voltages of the *d*-axis and *q*-axis, respectively; *i*_d_ and *i*_q_ represent the stator currents of the *d*-axis and *q*-axis, respectively; *L*_d_ and *L*_q_ represent the components of the stator inductance along the *d*-axis and *q*-axis, respectively; *R*_s_ is the stator winding resistance; *ω*_e_ is the motor electrical angular velocity; and *ψ*_f_ is the magnetic flux of the motor permanent magnet.

Among the common current control strategies for the servo permanent magnet drive system, the current control strategy with *i*_d_ = 0 simplifies the control structure by eliminating the *d*-axis current regulator, thereby reducing the computational burden. For the surface-mounted SPMSM adopted in this paper, it naturally ensures the maximum output torque per unit current, achieving high torque output and high efficiency. At the same time, the algorithm’s robustness has been verified through a large number of industrial applications, ensuring reliable operation under dynamic conditions. Therefore, this paper selects the current control strategy with *i*_d_ = 0. In the inverter modulation mode of the SVPWM, the instantaneous stator voltage has the following constraint relationship with the DC bus voltage *U*_dc_ of the servo system:

(2) $$\sqrt{u_{d}^{2}+u_{q}^{2}}\leq \frac{U_{\mathrm{dc}}}{\sqrt{3}}$$

Unlike the limitation of the stator voltage, the maximum value of the stator current is affected by multiple factors, mainly including the maximum output current of the inverter, the demagnetization current of the permanent magnet, the peak power duration, and the temperature rise limit of the motor windings. After considering all these factors comprehensively, the minimum value that meets all requirements is selected as the maximum stator armature current *i*_max_. At this time, the stator current of the motor satisfies the following constraint relationship:

(3) $$i_{d}^{2}+i_{q}^{2}\leq i_{\max}^{2}$$

The electromagnetic torque of the SPMSM can be expressed as

(4) $$T_{e}=\frac{3}{2}pi_{q}[i_{d}(L_{d}-L_{q})+\psi_{f}]$$

Generally, during the analysis and calculations, it is assumed that the *L*_d_ and *L*_q_ of the surface-mounted permanent magnet motor are equal. Moreover, due to the current control strategy of *i*_d_ = 0, combined with Equations (3) and (4), it can be determined that the maximum electromagnetic torque is directly proportional to the maximum stator current. At the same time, the maximum speed of the motor is, on the one hand, limited by the stator voltage equation, and the theoretical limit speed corresponds to the motor electromagnetic torque being 0 and the no-load reverse electromotive force approaching the speed value under the stator voltage. On the other hand, the mechanical stress of the motor rotor has a maximum speed limit, and, due to the load characteristics, the maximum speed value will also be set accordingly. When the maximum speed corresponding to other factors is higher than the theoretical limit speed, the maximum speed of the motor can be taken as the theoretical limit speed; otherwise, the speed under the corresponding limiting condition is taken as the maximum speed of the motor. At this time, the constraints of the maximum torque and the maximum speed can be expressed as follows.

The maximum torque and maximum speed of the SPMSM can be expressed as

(5) $$\left\{\begin{array}{l} \omega_{\mathrm{emax}}=\min(U_{\mathrm{dc}}/\sqrt{3}p\psi_{f},\omega_{\mathrm{mech}})\\ T_{\mathrm{emax}}=3pi_{\max}\psi_{f}/2 \end{array}\right.$$

where *ω*_mech_ represents the maximum speed limit determined by factors such as the motor rotor’s mechanical properties.

During the constant-power operation phase, the torque and speed of the motor are subject to the constraints of the stator voltage equation:

(6) $$\;{\left(\omega_{e}L_{q}i_{q}\right)}^{2}+{\left(R_{s}i_{q}+\omega_{e}L_{q}\frac{di_{q}}{dt}+\omega_{e}\psi_{f}\right)}^{2}\leq U_{\mathrm{dc}}^{2}/3$$

From Equations (5) and (6), it can be seen that the calculation of the torque and speed depends on parameters such as the winding resistance, stator winding *dq*-axis inductance, and permanent magnet flux linkage. The relevant electromagnetic parameters of the servo system will affect the boundary of the torque–speed trapezoidal feasible region formed by the maximum torque, constant-power curve, and maximum speed.

However, during actual operation, due to changes in the working environment’s temperature and the saturation degree of the motor, the above electromagnetic parameters exhibit nonlinear and time-varying characteristics. If the calculation of the trapezoidal feasible region boundary of the torque–speed gradient is based on the rated steady-state value and the dynamic characteristics of the trapezoidal feasible region boundary are ignored, errors in the setting of the dynamic boundary constraints will occur during actual operation, thereby causing the shortest positioning time obtained to exceed the operating limit of the servo system or failing to fully utilize the short-term output performance.

Therefore, in order to obtain the limit shortest response time of the SPMSM under actual operating conditions, and to provide data support for the quantification of the design margin for the short-term rapid response time of the servo system, it is necessary to accurately model and calculate the short-term dynamic trapezoidal feasible region boundary of the torque–speed gradient, so as to provide accurate short-term dynamic constraint conditions for the optimal trajectory planning of the continuous time of impact.

### 2.4. FEM Calculation of the Feasible Region of the Torque–Speed Trapezoid

Taking into account the nonlinearity and time-varying nature of the motor resistance, inductance, and magnetic flux parameters, this section uses the electromagnetic–thermal coupled FEM to calculate the boundary of the torque–speed trapezoidal feasible region of the SPMSM during short-term operation. Based on the finite element model of the prototype SPMSM, the following parameters are set: the DC bus voltage is 270 V, and the current control strategy selected is *i*_d_ = 0. Depending on the different temperatures of the motor stator winding, multiple sets of finite element models are established at equal intervals within the working temperature range of 0–70 °C. In each model set, the same maximum armature current of 9.5 A is set. Through the multi-field coupled finite element model, the data of the dynamic torque–speed trapezoidal feasible region boundary curves under different working temperatures and load conditions are obtained.

Following from the previous paragraph, although the maximum electromagnetic torque values of the motor and generator under the same current are the same in magnitude but opposite in sign, the damping effect of the friction torque causes the working curve of the SPMSM under trajectory planning to shift positively, thereby causing the torque–speed feasible region boundary curve of the motor working condition to trigger the dynamic constraint first. Therefore, without affecting the precision of the short-term dynamic feasible region boundary, in order to reduce the finite element calculation volume, only the torque–speed trapezoidal feasible region boundary of the motor working condition is solved.

**Figure 5:**
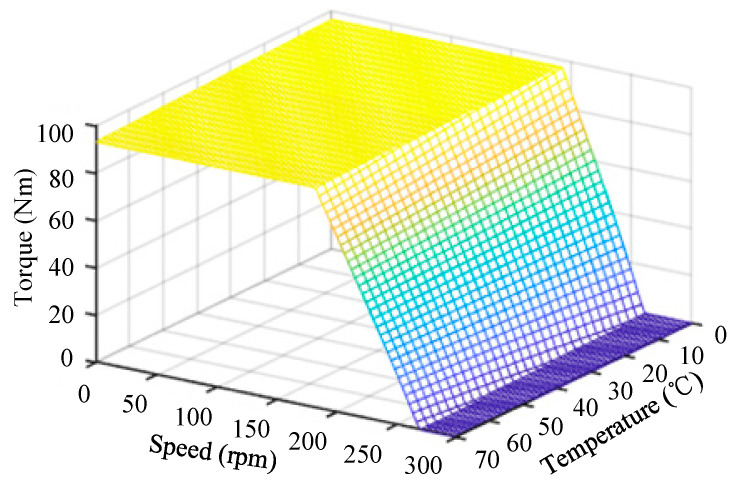
Boundary of the short-term dynamic trapezoid feasible region for torque–speed.

It should be emphasized that the above analysis holds when the trajectory planning is of the symmetrical type. When using asymmetric trajectory planning, the boundary of the generator working condition may still trigger the constraint first under a positive shift in the friction torque. The finite element calculation results regarding the short-term dynamic trapezoidal feasible region of the motor working condition are shown in Figure 5.

From Figure 5, it can be seen that the trapezoidal feasible region formed by the peak torque, constant-power curve, and maximum speed of the SPMSM motor under operating conditions changes with the temperature. When the motor operating temperature increases, the peak torque will decrease accordingly. The peak torque at 70 °C is 3.63% lower than that at 0 °C, and the peak speed also decreases by 6.67%. At the same time, due to the influence of magnetic saturation, the torque–current ratio coefficient under the peak current is also lower than the rated operating condition. The above results further indicate that the traditional method of setting dynamic constraints based on linear non-time-varying torque–speed boundary parameters inevitably leads to the solution of the ultimate shortest positioning time exceeding or failing to fully utilize the short-term output performance of the SPMSM. This fully validates the necessity of considering the nonlinearity and time-varying nature of the parameters under short-term dynamic operation.

Therefore, in order to fully utilize the short-term overload capacity of the SPMSM, it is necessary to consider the average heat power of the continuous trajectory on the basis of the trapezoidal feasible region boundary of the torque–speed and directly use the heat power constraint as the standard for selecting the torque boundary, so that the torque of the impact continuous trajectory can also exceed the peak torque in the short term and operate, thereby further reducing the positioning time.

## 3. Time-Optimal Trajectory Planning Algorithm Based on Dynamic Feasible Region Constraints

### 3.1. Optimization Objectives and Constraints

When the start and end times and positions are set, and the speed and acceleration are both 0 at the start and end moments, the six polynomial coefficients of the quintic polynomial trajectory function can be uniquely determined by six sets of kinematic constraints. Based on the quintic polynomial trajectory function, the expressions for the position, velocity, and acceleration are as follows:

(7) $$\left\{\begin{array}{l} \theta (t)=\frac{10D}{T_{0}^{3}}t^{3}-\frac{15D}{T_{0}^{4}}t^{3}+\frac{6D}{T_{0}^{5}}t^{3}\\ \omega (t)=\frac{30D}{T_{0}^{3}}t^{2}-\frac{60D}{T_{0}^{4}}t^{3}+\frac{30D}{T_{0}^{5}}t^{4}\\ \alpha (t)=\frac{60D}{T_{0}^{3}}t-\frac{180D}{T_{0}^{4}}t^{2}+\frac{120D}{T_{0}^{5}}t^{3} \end{array}\right.$$

where *D* represents the target positioning angle, and *T*_0_ denotes the total time for the positioning process.

Under short-term and rapid positioning conditions, the SPMSM needs to quickly complete the positioning based on the target angle instruction given by the system. Within the travel range of 0–180°, each angle corresponds to a specific limit shortest positioning time. The optimization objective of the five-degree-of-freedom polynomial time-optimal trajectory planning is to optimize and calculate the shortest positioning time according to different positioning angles, while considering the changes in the dynamic boundaries under different working temperatures. Ultimately, a mapping relationship between the shortest time, working temperature, and positioning angle needs to be established.

Under the six sets of kinematic constraints on the position, velocity, and acceleration at the start and end times, the six polynomial coefficients of the time-optimal trajectory planning function can be calculated based on the target positioning angle *D* and positioning time *T*_0_. The explicit form of the linear equations corresponding to these six sets of boundary constraints can be expressed as follows:

(8) $$\left[\begin{array}{ccccc} 1 & 0 & 0 & \ldots & 0\\ 0 & 1 & 0 & \ldots & 0\\ 0 & 0 & 2 & \ldots & 0\\ 1 & T_{0} & T_{0}^{2} & \ldots & T_{0}^{5}\\ 0 & 1 & 2T_{0} & \ldots & 5T_{0}^{4}\\ 0 & 0 & 2 & \ldots & 20T_{0}^{3} \end{array}\right]a=\left[\begin{array}{c} 0\\ 0\\ 0\\ D\\ 0\\ 0 \end{array}\right]$$

where $a={\left[a_{0},a_{1},\cdots ,a_{5}\right]}^{T}$ is the vector of polynomial coefficients, *M* is the constraint matrix composed of time exponents and factorials, and $b={\left[0,0,0,D,0,0\right]}^{T}$ is the boundary value vector.

For different positioning angles, the corresponding shortest positioning time is uniquely determined by the dynamic constraints, and the dynamic constraints include the boundary values of the velocity, acceleration, and impact. The impact dynamic constraints are as follows:

(9) $$j(t)=\frac{60D}{T_{0}^{3}}a-\frac{360D}{T_{0}^{4}}t+\frac{360D}{T_{0}^{5}}t^{2}\leq j_{\max}$$

where the maximum impact value is determined by the servo system based on the load characteristics.

Based on the FEM electromagnetic–thermal coupling calculation, it can be determined that, for the SPMSM prototype operating at an effective armature current of 9.5 A and a peak rotational speed of 186 rpm, the total power loss of the motor at this time is 720 W. Therefore, in the short-term rapid positioning operation condition of the SPMSM, as long as the average thermal power during a single instance of rapid positioning does not exceed 720 W, it is possible to ensure that, when its working curve torque reaches the peak torque, the fifth-degree polynomial still meets the temperature rise limit requirements of the motor.

Converting the speed and acceleration into the rotational speed and electromagnetic torque, and substituting them into the energy consumption model [25], the energy consumed by the single positioning of the fifth-degree polynomial can be calculated, thereby setting the average power constraint *P*_av_ as follows:

(10) $$P_{\mathrm{av}}=E/T_{0}=\frac{1}{T_{0}}\int_{0}^{T_{0}}\left(f(T,n,T_{e})+\frac{\pi n\cdot T_{e}}{30}\right)dt\leq 720$$

**Figure 6:**
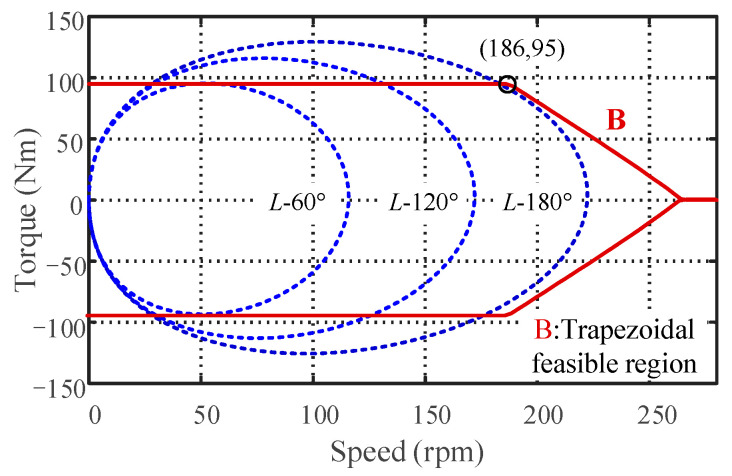
Short-term dynamic feasible region constraints for torque and speed.

The maximum acceleration is limited by the average power of short-term overload, and the maximum speed is determined by the FEM calculation data for the trapezoidal feasible region. The above three groups of dynamic boundaries jointly form the proposed short-term dynamic feasible region constraint boundary. The operating range for the torque and speed in this short-term dynamic feasible region has been significantly expanded compared to using the rectangular feasible region and the trapezoidal feasible region. The short-term dynamic feasible region is shown in Figure 6.

From Figure 6, it can be seen that the working curve corresponding to the trajectory planning can exceed the limit of the peak working point. When the positioning angle is large, the working curve will reach the constraint of the constant-power curve. As the positioning angle decreases, the torque and speed required for the trajectory will also decrease. At the same time, due to the constraint of the average thermal power, even if the torque and speed exceed the rectangular range of the peak working point, it will not cause the motor temperature to exceed the limit. However, it should be emphasized that the shortest time trajectory under any angle still needs to meet the constraint of the maximum impact value to avoid the situation where the torque and speed are not beyond the feasible range but the rate of torque change is too fast.

Therefore, the dynamic constraint based on the short-term dynamic feasible region of the torque and speed can be expressed as follows:

(11) $$\left(\omega (t),\alpha (t))\in g(T,n,T_{e}),\forall t\in [0,T_{0}\right]$$

Among the terms, *g*(*T*, *n*, *T*_e_) represents the dynamic feasible region of the torque–speed and the conversion function between speed and acceleration.

The kinematic and dynamic constraints of the combined Equations (8)–(11) can transform the time-optimal trajectory planning problem into a minimization problem with both equality and nonlinear constraints:

(12) $$\left\{\begin{array}{l} \min T_{0}\\ s.t.Ma=b,P_{\mathrm{av}}\leq 720,j(t)\leq j_{\max}\\ \left(\omega (t),\alpha (t))\in g(T,n,T_{e}\right) \end{array}\right.$$

### 3.2. Time-Optimal Trajectory Solving Algorithm Based on SLSQP

Considering that nonlinear dynamic constraints are ubiquitous in time-optimal planning, the Sequential Least Squares Quadratic Programming (SLSQP) method can achieve a better balance between the handling of nonlinear constraints and solution efficiency. Therefore, the SLSQP algorithm is selected. The solution algorithm flow for time-optimal trajectory planning based on fifth polynomial is shown in Table 2.

**Figure 7:**
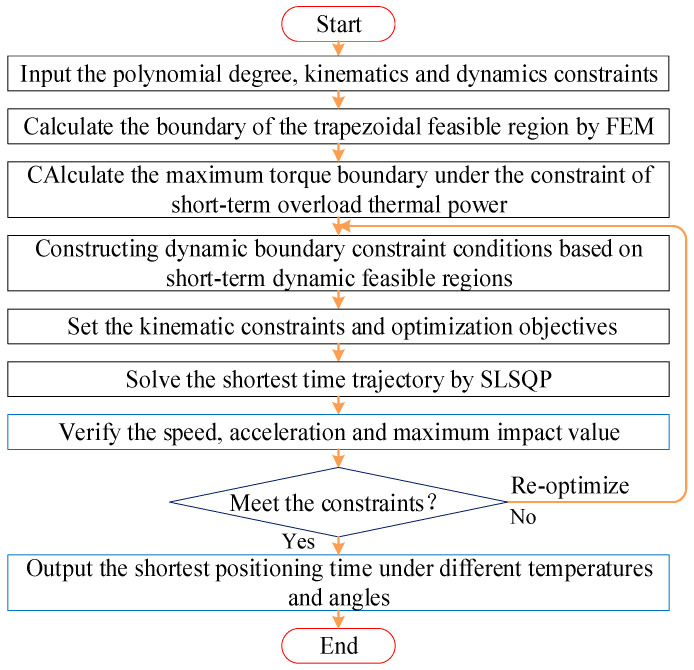
Time-optimal trajectory optimization design process based on short-term dynamic feasible region constraints.

Considering the requirement of the photoelectric pod load for the continuity of impact, and the comparison of the complexity of the trapezoidal, S-curve, and quintic polynomial trajectory functions, the quintic polynomial is selected as the target trajectory. Based on the dynamic constraint conditions of the short-term dynamic feasible region, the time-optimal trajectory of the quintic polynomial is optimized and solved. At the same time, the method of offline calibration of the shortest positioning time under different temperatures and angles, and the online application of its fitting model, is proposed to reduce the trajectory calculation volume under the online millisecond-level response. The overall optimization design process is shown in Figure 7. The detailed steps are as follows:

Firstly, input the polynomial degree and kinematic and dynamic constraints; based on the FEM calculation of the trapezoidal feasible region of the torque and speed, the boundary data of the constant-power and maximum speed curves are obtained. According to the maximum thermal power value of the SPMSM under peak operation conditions, the maximum torque constraint of the integrated energy consumption model is set, and the dynamic boundary constraint conditions based on the short-term dynamic feasible region are constructed. Secondly, according to the requirements of the actual positioning conditions, the kinematic constraints of the quintic polynomial trajectory are set, and the corresponding optimization design objectives and variables are selected. The SLSQP constrained optimization algorithm is used to solve the time-optimal trajectory planning function. Then, the speed, acceleration, and impact values of the optimized time-optimal trajectory are calculated, and the satisfaction of all constraints is verified. If satisfied, the corresponding shortest time data are output; if not, the optimization is re-performed. Finally, based on the offline calibration data of the quintic polynomial shortest positioning time under different temperatures and positioning angles obtained from the optimization, the corresponding shortest time fitting model is established to be applied in online operation.

### 3.3. Calibration of the Shortest Positioning Time

In the actual short-term positioning condition of the servo system, after the motor controller receives the target positioning angle given by the system, it is necessary to enable the SPMSM to achieve a rapid response within milliseconds. This makes it difficult to conduct online optimization and solution for optimal trajectory planning within a short period of time based on the computing power of the DSP chip. Based on the optimization solution algorithm described in Section 3.2, the shortest positioning time under different target angles and working temperatures can be obtained. Combining Equation (7), it can be found that any quintic polynomial trajectory can be uniquely determined by the positioning time and angle under kinematic constraints. Moreover, the calculation amount required to solve the polynomial coefficients based on the DSP is relatively small. Therefore, an offline calibration method for the shortest positioning time is proposed. Considering that the mapping relationship has fewer input and output variables, a mathematical analytical expression is used to fit the mapping relationship, and the fitting correlation coefficient *R*^2^ is controlled to be greater than 0.95 to balance the data fitting accuracy.

**Figure 8:**
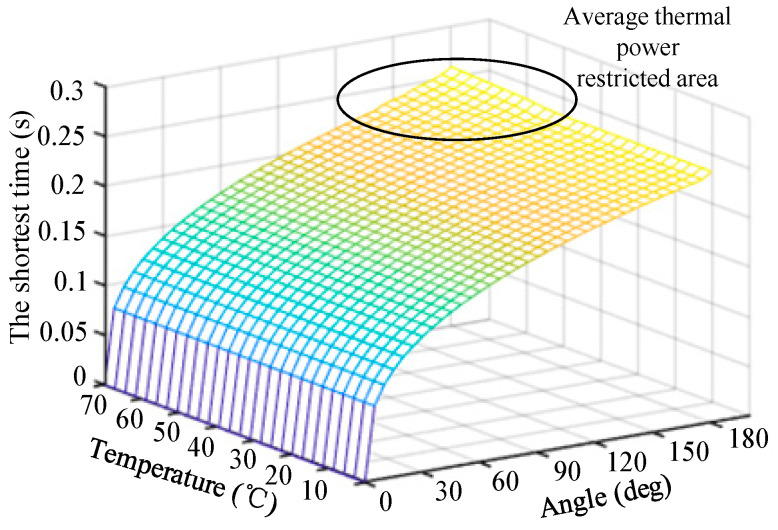
The shortest positioning time under different positioning angles and working temperatures.

Within the working temperature range of 0–70 °C and the positioning angle range of 0–180°, the target angles and working temperatures of the SPMSM were traversed at equal intervals, and the data matrix of the shortest positioning time under different angles and working temperatures was obtained. Using the optimization algorithm from the previous section, the calculated shortest time results were obtained, as shown in Figure 8.

As shown in Figure 8, based on the dynamic constraints of the proposed short-term dynamic feasible region, the shortest positioning time of the quintic polynomial trajectory decreases as the positioning angle decreases. However, as the target angle further decreases, the reduction in the shortest positioning time gradually increases. At the same time, due to the changes in the loss characteristics of the SPMSM under different working temperatures, the average thermal power for rapid positioning gradually approaches the maximum constraint value of 720 W as the working temperature increases. The shortest positioning time of 180° at 70 °C is 2.77% longer than that at 35 °C. This is consistent with the analysis results for the trapezoidal feasible region of the torque and speed obtained from the FEM calculations under different working temperatures in the previous section. This further indicates the necessity of considering the dynamic changes in the dynamic boundary constraints in the time-optimal trajectory planning of SPMSMs.

**Figure 9:**
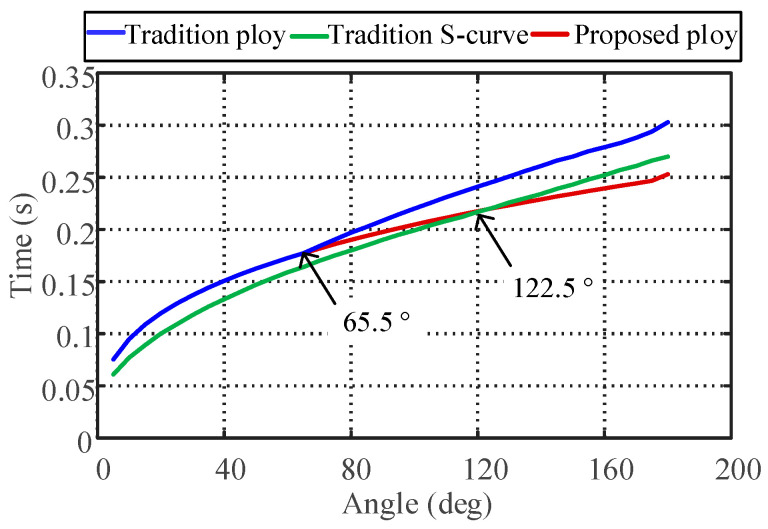
Comparison of the shortest positioning times for the three schemes within the 0–180° range.

In order to comprehensively compare the time optimization effect of the proposed dynamic constraint method to that of the traditional method within the 0–180° travel range, the above three sets of dynamic constraints and trajectory combination schemes were adopted to scan and optimize all angles within the travel range, and the shortest positioning time data at different angles were obtained. After a large number of optimization calculations, within the 0–180° positioning range, the shortest positioning times of the proposed dynamic constraint + quintic polynomial trajectory, the traditional dynamic constraint + S-curve trajectory, and the traditional dynamic constraint + quintic polynomial trajectory are as shown in Figure 9.

From Figure 9, it can be seen that, within the positioning angle range of 122.5° to 180°, the shortest positioning time of the proposed constraint + quintic polynomial trajectory is shorter than those of both the S-curve trajectory and the quintic polynomial trajectory under traditional constraints. When the positioning angle is less than 122.5°, the positioning time of the traditional constraint + S-curve trajectory begins to be lower than that of the proposed constraint + quintic polynomial trajectory. When the positioning angle further decreases to 65.5°, the positioning time of the proposed constraint + quintic polynomial trajectory is the same as that of the traditional constraint + quintic polynomial trajectory. The above results indicate that, in the case of a large travel angle, the dynamic constraint method based on the short-time dynamic feasible region proposed in this paper can ensure the continuity of short-time positioning impact changes, and the positioning time is shorter than that of the S-curve trajectory. This can effectively improve the short-time response speed of the SPMSM in large travel situations. Within the range of approximately 2/3 of the positioning travel, the shortest positioning time is better than that of the quintic polynomial trajectory under traditional dynamic constraints, effectively enhancing the dynamic response capabilities of the SPMSM for photoelectric pods in short-time rapid positioning conditions.

## 4. Experimental Verification

### 4.1. Prototype Test Platform

**Figure 10:**
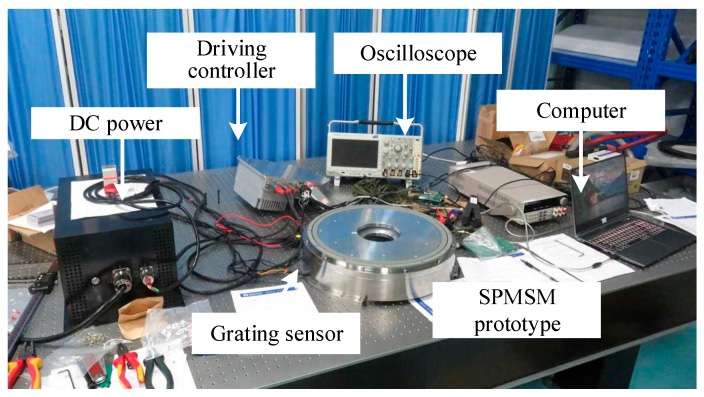
Time-optimal trajectory positioning time test platform.

To further validate the optimization effect of the time-optimal trajectory planning under the short-time dynamic feasible region constraint proposed in this paper, time-optimal trajectory planning tests under different positioning angles and operating temperatures were conducted based on the developed SPMSM prototype. The constructed positioning time test platform is shown in Figure 10. In the experimental tests, the time-optimal trajectory planning method was given by the host computer, and the trajectory planning function was used as the position-loop reference signal. The control chip employed was a DSP TMS320-F28377, and the motor current control algorithm adopted *i*_d_ = 0. The PI parameters of the three loops were adjusted according to different positioning conditions, but, under the same operating conditions, the same PI tuning parameters were used for the three trajectory planning schemes to ensure fairness in the comparison of the minimum positioning time. The voltage and current signals were obtained through the oscilloscope and probes. The position and speed signals were obtained through the absolute optical grating encoder (model RESM20U, Renishaw); its grating pitch was 20, according to the manufacturer’s datasheet. After considering the noise impact caused by electronic subdivision errors, the system accuracy could reach ±0.5 arc seconds.

### 4.2. Positioning Time Tests Under Different Positioning Angles

Based on the turning angles corresponding to the minimum positioning times of the three trajectory groups, positioning angles of 180°, 122.5°, and 65.5° were selected. Meanwhile, three trajectory schemes were adopted for experimental testing at a motor operating temperature of 35 °C, namely the S-curve and the quintic polynomial under traditional dynamic constraints, as well as the quintic polynomial under the proposed dynamic constraints.

(1) Experiment on time-optimal trajectory planning at a positioning angle of 180°

**Figure 11:**
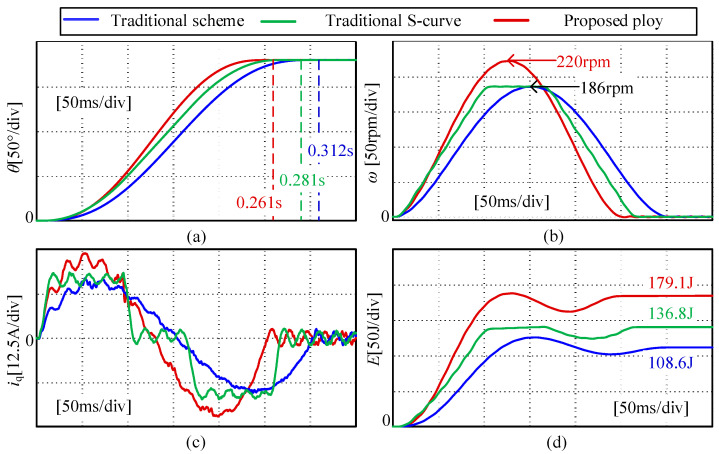
The experimental waveforms for 3 groups of trajectories under 180° positioning: (**a**) waveforms of position; (**b**) waveforms of velocity; (**c**) waveforms of *i*_q_; (**d**) waveforms of energy.

Under a positioning angle of 180°, the corresponding test waveforms of the position, velocity, *i*_q_, and energy for the three trajectory groups—namely, the proposed short-time dynamic feasible region constraint + quintic polynomial trajectory, the traditional dynamic constraint + S-curve trajectory, and the traditional dynamic constraint + quintic polynomial trajectory—are shown in Figure 11.

As shown in Figure 11a, when the motor positioning accuracy reaches within the ±0.05° error band of the target angle, the positioning times of the proposed constraint + quintic polynomial, traditional constraint + S-curve, and traditional constraint + quintic polynomial are 0.261 s, 0.281 s, and 0.312 s, respectively. Compared with the traditional S-curve and quintic polynomial, the proposed method reduces the positioning time by 7.12% and 16.3%, respectively. The relative errors between the experimental test times and the simulation times are 3.16%, 4.07%, and 2.97%, respectively. From Figure 11b, it can be seen that the maximum speed of the proposed constraint + quintic polynomial exceeds the peak speed of 186 rpm, while the maximum speeds of the S-curve and quintic polynomial under traditional constraints are both within the peak speed constraint range. From Figure 11c, the *i*_q_ current of the proposed constraint + quintic polynomial is higher than those of the other two trajectory schemes; the *i*_q_ current of the S-curve under traditional constraints is higher than that of the quintic polynomial, and the *i*_q_ current of the traditional constraint + S-curve runs at the peak current for a certain period, which is consistent with the previous simulation analysis. From Figure 11d, the positioning energy consumption of the proposed constraint + quintic polynomial, traditional constraint + S-curve, and traditional constraint + quintic polynomial is 179.1 J, 136.8 J, and 108.6 J, respectively, with errors of 5.2%, 6.65%, and 4.97% compared to the previous simulated energy consumption values. At this point, the average thermal power of the proposed constraint + quintic polynomial is 686 W, which is lower than the short-term overload thermal power constraint value of 720 W.

(2) Experiment on time-optimal trajectory planning at a positioning angle of 122.5°

**Figure 12:**
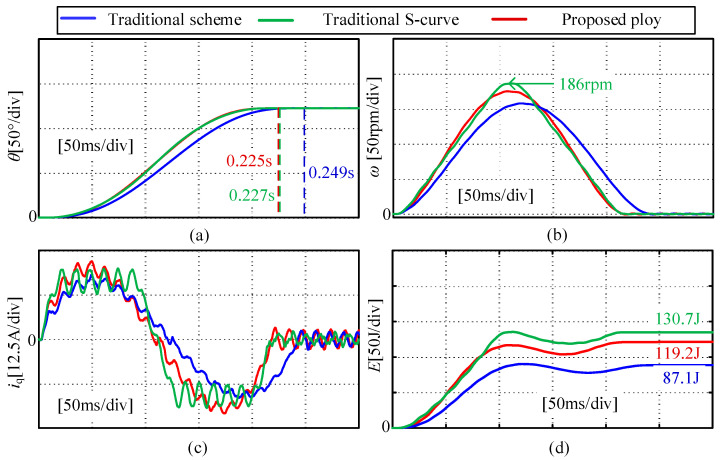
The experimental waveforms for 3 groups of trajectories under 122.5° positioning: (**a**) waveforms of position; (**b**) waveforms of velocity; (**c**) waveforms of *i*_q_; (**d**) waveforms of energy.

Under a positioning angle of 122.5°, the corresponding test waveforms of the position, velocity, *i*_q_, and energy for the three trajectory groups—namely, the proposed short-time dynamic feasible region constraint + quintic polynomial trajectory, the traditional dynamic constraint + S-curve trajectory, and the traditional dynamic constraint + quintic polynomial trajectory—are shown in Figure 12.

As shown in Figure 12a, when the motor positioning accuracy reaches within the ±0.05° error band of the target angle, the positioning times of the proposed constraint + quintic polynomial, traditional constraint + S-curve, and traditional constraint + quintic polynomial are 0.225 s, 0.227 s, and 0.249 s, respectively. Compared with the traditional S-curve, the proposed method reduces the positioning time by 0.88%, and the two groups have nearly identical times; compared with the traditional quintic polynomial, the reduction is 9.64%. The relative errors between the experimental test times and the simulation times are 2.74%, 3.65%, and 2.47%, respectively. From Figure 12b, it can be seen that the speed of the S-curve under traditional constraints is the highest, but it does not run continuously at the maximum speed; rather, it stays at the turning point of the maximum speed. The maximum speeds of the proposed constraint + quintic polynomial and the traditional constraint + quintic polynomial decrease in order. From Figure 12c, the *i*_q_ current of the proposed constraint + quintic polynomial is higher than those of the other two trajectory schemes; the *i*_q_ current of the S-curve under traditional constraints runs at its maximum value for a certain period, while the current of the traditional constraint + quintic polynomial is the smallest. From Figure 12d, the positioning energy consumption of the proposed constraint + quintic polynomial, traditional constraint + S-curve, and traditional constraint + quintic polynomial is 119.2 J, 130.7 J, and 87.1 J, respectively. The energy consumption of the traditional constraint + S-curve trajectory is the highest. The average thermal power of the proposed constraint + quintic polynomial is 529.7 W, which is lower than the short-term overload thermal power constraint value of 720 W.

(3) Experiment on time-optimal trajectory planning at a positioning angle of 65.5°

**Figure 13:**
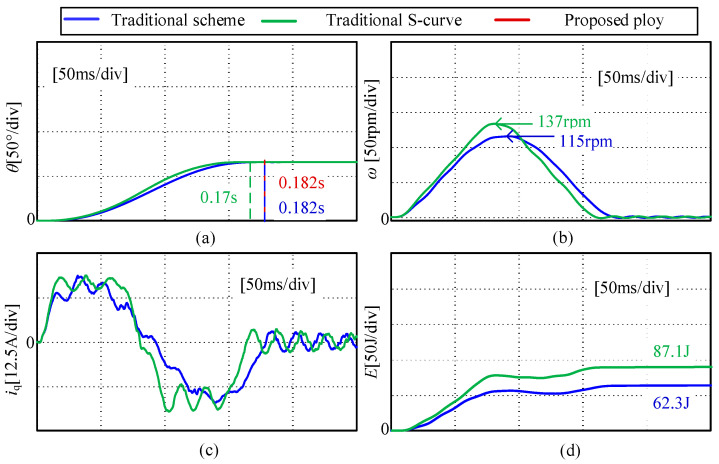
The experimental waveforms for 3 groups of trajectories under 65.5° positioning: (**a**) waveforms of position; (**b**) waveforms of velocity; (**c**) waveforms of *i*_q_; (**d**) waveforms of energy.

Under a positioning angle of 65.5°, the corresponding test waveforms of the position, velocity, *i*_q_, and energy for the three trajectory groups—namely, the proposed short-time dynamic feasible region constraint + quintic polynomial trajectory, the traditional dynamic constraints + S-curve trajectory, and the traditional dynamic constraints + quintic polynomial trajectory—are shown in Figure 13.

As shown in Figure 13a, when the motor positioning accuracy reaches within the ±0.05° error band of the target angle, the positioning times of the proposed constraint + quintic polynomial, traditional constraint + S-curve, and traditional constraint + quintic polynomial are 0.182 s, 0.17 s, and 0.182 s, respectively. Compared with the traditional S-curve, the proposed method increases the positioning time by 7%, and it is the same as that of the traditional constraint + quintic polynomial trajectory. The relative errors between the experimental test times and the simulation times are 2.25%, 3.03%, and 2.25%, respectively. From Figure 13b, it can be seen that the maximum speeds of the three trajectory groups do not reach the peak speed; the speed of the traditional constraint + S-curve is the highest, and the speed curves of the quintic polynomial trajectories under the proposed constraint and the traditional constraint are the same. From Figure 13c, the *i*_q_ currents of the quintic polynomial trajectories under the proposed constraint and the traditional constraint are the same, and the *i*_q_ current of the traditional constraint + S-curve trajectory is higher than those of the other two trajectories. From Figure 13d, the positioning energy consumption of the proposed constraint + quintic polynomial, traditional constraint + S-curve, and traditional constraint + quintic polynomial is 62.3 J, 87.1 J, and 62.3 J, respectively. At this point, the average thermal power of the proposed constraint + quintic polynomial is 342.3 W, which is lower than the short-term overload thermal power constraint value of 720 W.

From the analysis of the experimental waveforms at the three different positioning angles above, it can be seen that, due to the discontinuity of jerk, the S-curve results in significantly higher fluctuations in speed, current, and energy than the two quintic polynomial trajectories, demonstrating the effectiveness of using the quintic polynomial trajectory with continuous jerk.

A comparison of the experimental test results regarding the time required for the three combinations of dynamic constraints and trajectory types at the boundary positioning angles is shown in Table 3, along with the percentage of time saved by the proposed dynamic constraint + quintic polynomial compared with the traditional dynamic constraint + quintic polynomial trajectory.

As can be seen from Table 3, the parameters of the three trajectory groups are generally consistent with the simulation analysis results. The simulation and experimental errors for parameters such as the positioning time and positioning energy consumption essentially meet the practical engineering requirements. This further demonstrates that the proposed dynamic method with short-time dynamic feasible region constraints can effectively reduce the positioning time under large positioning strokes, and it even achieves a shorter positioning time than the traditional S-curve trajectory in some angle ranges. Compared with the traditional quintic polynomial, the proposed method achieves a reduction in positioning time over a larger range of strokes.

### 4.3. Positioning Time Tests Under Different Working Temperatures

**Figure 14:**
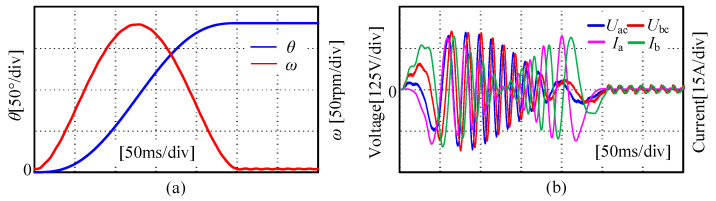
The waveforms for the positioning experiment with a 0.253 s trajectory at 35 °C: (**a**) waveform of position and velocity; (**b**) waveform of voltage and current.

**Figure 15:**
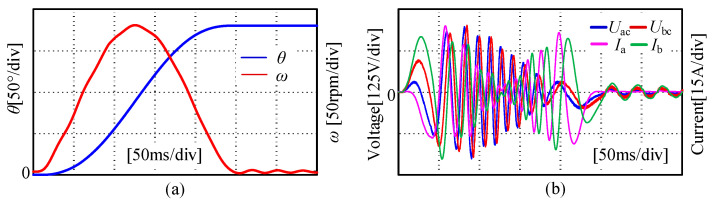
The waveforms for the positioning experiment with a 0.253 s trajectory at 70 °C: (**a**) waveform of position and velocity; (**b**) waveform of voltage and current.

**Figure 16:**
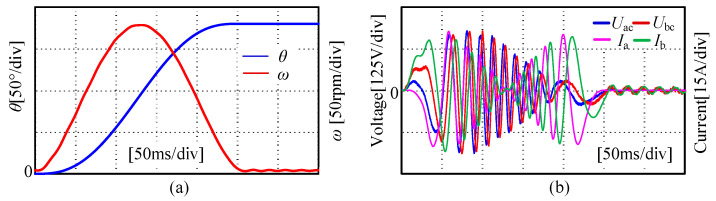
The waveforms for the positioning experiment with a 0.26 s trajectory at 70 °C: (**a**) waveform of position and velocity; (**b**) waveform of voltage and current.

Since traditional dynamic constraint methods do not consider the influence of the temperature on the torque–speed operating boundaries, this section describes a comparative test of the positioning energy consumption under different operating temperatures for the quintic polynomial trajectory under the proposed constraint. A positioning angle of 180° is selected, with operating temperatures of 35 °C and 70 °C. The positioning energy consumption is tested at both 35 °C and 70 °C using a quintic polynomial trajectory with a minimum positioning time of 0.253 s. Then, a trajectory with a minimum positioning time of 0.26 s is used to test the positioning energy consumption at 70 °C. The combined test results of the three trajectory-and-temperature groups are shown in Figure 14, Figure 15 and Figure 16.

As shown in Figure 14, Figure 15 and Figure 16, under the same positioning time, when the operating temperature increases from 35 °C to 70 °C, the voltage and current waveforms rise significantly, reflecting that an increase in temperature reduces the output torque of the SPMSM, thus requiring higher voltage and current outputs. On the other hand, increasing the positioning time at 70 °C can effectively reduce the required torque and speed, further lowering the amplitudes of the voltage and current waveforms. This fully demonstrates the necessity of considering the time-varying characteristics of dynamic constraints under different operating temperatures.

**Figure 17:**
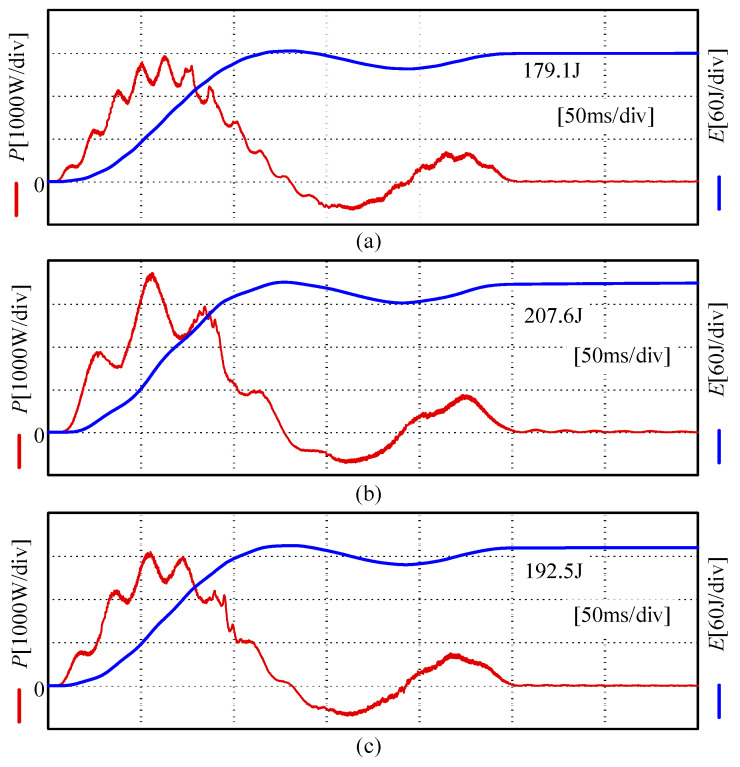
Power and energy consumption test waveforms for the three sets of trajectories: (**a**) 0.253 s positioning at 35 °C; (**b**) 0.253 s positioning at 70 °C; (**c**) 0.26 s positioning at 70 °C.

Based on the two-wattmeter method for power calculation in three-phase circuits, the instantaneous power waveforms of the above three trajectory groups were obtained and integrated in real time to calculate the energy consumption, as shown in Figure 17.

As can be seen from Figure 17, under the same positioning angle and positioning time, as the operating temperature increases, the positioning energy consumption also increases from 179.1 J at 35 °C to 207.6 J, indicating that the copper loss of the motor increases with the temperature, and the energy consumed to complete the same trajectory planning also increases accordingly. At this point, the average thermal power of the SPMSM increases from 686 W to 792 W, which already far exceeds the set maximum thermal power constraint. Therefore, the positioning time must be controlled to reduce the average thermal power. After increasing the positioning time, the positioning energy consumption decreases to 192.5 J, and the average thermal power at this time is 718 W, which is approaching the thermal power constraint value. Due to the energy feedback during the deceleration process, the energy consumption waveform undergoes a downward trend after rising.

The above experimental results fully verify that, when setting the dynamic constraints for time-optimal trajectory planning, the influence of changes in operating temperature on the minimum positioning time must be considered. The dynamic method based on the short-time dynamic feasible region constraint proposed in this paper not only effectively shortens the minimum positioning time but also takes into account the limitation of the heat generation power under actual motor operating conditions, providing an effective approach to maximizing the dynamic response capabilities of the SPMSMs used in photoelectric pods under short-term rapid positioning conditions.

## 5. Conclusions

To address the problem whereby existing dynamic boundary conditions for servo motors cannot accurately characterize the short-time dynamic feasible region of the torque and speed under short-term high overload, a wide temperature range, and non-periodic transient conditions, and thus cannot fully utilize the short-term output performance of the SPMSM, this paper proposes a time-optimal trajectory planning method under short-time dynamic feasible region constraints. Compared with existing studies, this research shows that it is possible to effectively enhance the utilization rate of the motor’s output performance during short-term operation. The main conclusions are as follows:

(1) Considering the parameter nonlinearity and time-varying characteristics, an FEM model is established to obtain accurate feasible region boundary data. Based on the torque constraint method, using the short-term overload thermal average power as the limit, a torque–speed short-time dynamic feasible region constraint method is proposed, in which both the maximum torque and maximum speed break through the boundary constraints of the traditional rectangular feasible region
(2) A time-optimal trajectory solution algorithm based on SLSQP is proposed. The minimum positioning time under different operating temperatures and positioning angles with the short-time dynamic feasible region constraint is calibrated offline. The simulation analysis shows that, within the range of 122.5° to 180°, the positioning time of the proposed method is shorter than that in both the traditional S-curve and quintic polynomial trajectories, achieving a maximum reduction of 6.3% and 16.5%, respectively.
(3) The time-optimal trajectory under different positioning angles and operating temperatures is verified on an experimental platform. The results show that the proposed dynamic constraint method achieves a positioning time that is shorter than that of the S-curve by nearly one-third of the stroke range. Compared with the quintic polynomial under traditional constraints, the positioning time is reduced by over two-thirds of the stroke range. Moreover, at large stroke angles, time savings of 7.12% and 16.3% are achieved. The results also demonstrate that the influence of changes in operating temperature on the minimum positioning time must be considered when setting dynamic constraints.

## Figures and Tables

**Table 1:** Main parameters of SPMSM.

Parameter	Angle	Time	Maximum Speed	Maximum Acceleration	Maximum Impact
Value	180°	0.4 s	843.75 deg/s	6493 deg/s^2^	168,750 deg/s^3^

**Table 2:** The solution algorithm flow for time-optimal trajectory planning.

Algorithm: Time-Optimal Trajectory Calculation Process
Input: Polynomial degree, kinematic and dynamic constraints
Output: The time-optimal trajectory function, minimum time at different angles and temperatures
1: Initialize the polynomial coefficient vector *a* = [*a*_0_ *a*_1_ … *a*_5_]
2: Define boundary condition constraints: [*θ*(0) *ω*(0) *α*(0)] and [*θ*(*t*_f_) *ω*(*t*_f_) *α*(*t*_f_)]
3: For Poly_coeffs (*a*) = 0, 1, …, 5. Set the optimization objective function
4: Calculate the corresponding velocity, acceleration, and impact function of the trajectory based on the initial polynomial coefficients. Verify whether the dynamic boundary constraints are satisfied and return the positioning time.
5: Save the coefficient vector *a* and *T*_0_
6: Update coefficient matrix values
7: Use SLSQP to solve result = min(objective, Poly_coeffs, constraints)
8: Whether to obtain the coefficient vector with the smallest time value
end for 9: Output the result of the minimum polynomial

**Table 3:** Time test of different dynamic constraint methods under different angles.

Positioning Time	180°	122.5°	65.5°
Traditional scheme (s)	0.312	0.249	0.182
Traditional S-curve (s)	0.281	0.227	0.17
Proposed scheme (s)	0.261	0.225	0.182
Time saving percentage	16.3%	9.64%	0%

## Data Availability

The data presented in this study are available upon request from the corresponding author. The data are not publicly available due to the confidentiality restrictions of this study.
